# Odorant Receptors for Detecting Flowering Plant Cues Are Functionally Conserved across Moths and Butterflies

**DOI:** 10.1093/molbev/msaa300

**Published:** 2020-11-24

**Authors:** Mengbo Guo, Lixiao Du, Qiuyan Chen, Yilu Feng, Jin Zhang, Xiaxuan Zhang, Ke Tian, Song Cao, Tianyu Huang, Emmanuelle Jacquin-Joly, Guirong Wang, Yang Liu

**Affiliations:** 1 State Key Laboratory for Biology of Plant Diseases and Insect Pests, Institute of Plant Protection, Chinese Academy of Agricultural Sciences, Beijing, China; 2 Guangdong Laboratory for Lingnan Modern Agriculture (Shenzhen Branch), Genome Analysis Laboratory of the Ministry of Agriculture, Agricultural Genomics Institute at Shenzhen, Chinese Academy of Agricultural Sciences, Shenzhen, China; 3 INRAE, Sorbonne Université, CNRS, IRD, UPEC, Université de Paris, Institute of Ecology and Environmental Sciences of Paris, Versailles, France

**Keywords:** odorant receptor, *Helicoverpa armigera*, Glossata, plant volatile, phenylacetaldehyde

## Abstract

Odorant receptors (ORs) are essential for plant–insect interactions. However, despite the global impacts of Lepidoptera (moths and butterflies) as major herbivores and pollinators, little functional data are available about Lepidoptera ORs involved in plant-volatile detection. Here, we initially characterized the plant-volatile-sensing function(s) of 44 ORs from the cotton bollworm *Helicoverpa armigera*, and subsequently conducted a large-scale comparative analysis that establishes how most orthologous ORs have functionally diverged among closely related species whereas some rare ORs are functionally conserved. Specifically, our systematic analysis of *H. armigera* ORs cataloged the wide functional scope of the *H. armigera* OR repertoire, and also showed that HarmOR42 and its *Spodoptera littoralis* ortholog are functionally conserved. Pursuing this, we characterized the HarmOR42-orthologous ORs from 11 species across the Glossata suborder and confirmed the HarmOR42 orthologs form a unique OR lineage that has undergone strong purifying selection in Glossata species and whose members are tuned with strong specificity to phenylacetaldehyde, a floral scent component common to most angiosperms. In vivo studies via HarmOR42 knockout support that HarmOR42-related ORs are essential for host-detection by sensing phenylacetaldehyde. Our work also supports that these ORs coevolved with the tube-like proboscis, and has maintained functional stability throughout the long-term coexistence of Lepidoptera with angiosperms. Thus, beyond providing a rich empirical resource for delineating the precise functions of *H. armigera* ORs, our results enable a comparative analysis of insect ORs that have apparently facilitated and currently sustain the intimate adaptations and ecological interactions among nectar feeding insects and flowering plants.

## Introduction

Insects usually use plants for food and shelter, and their interactions are essential for the entire terrestrial ecosystem (Schuman and Baldwin 2016; Guo et al. 2020). Chemical cues, which are mainly detected by chemoreceptors, have been reported to play important roles in multiple ecological behaviors of insects such as mating, foraging, and oviposition (Schoonhoven et al. 2005; Joseph and Carlson 2015). Among these cues, olfactory ones dominate the host finding process and participate, together with contact cues, in host evaluation and final acceptation (Bruce et al. 2005; Xu and Turlings 2018). The insect olfactory process is mediated by a highly efficient and precise detection system at the core of which are the odorant receptors (ORs) (Fleischer et al. 2018). With the help of genomic information and powerful genetic tools, ORs were first identified in the insect model *Drosophila melanogaster* and their functions have been extensively studied (Clyne et al. 1999; Gao and Chess 1999; Vosshall et al. 1999; Hallem and Carlson 2006). In the last two decades, repertoires of OR sequences have been characterized in numerous species due to transcriptome or genome sequencing. The number of ORs in these collections varies considerably, from ten in the body louse *Pediculus humanus humanus* to up to 400 in the ants *Camponotus floridanus* and *Harpegnathos saltator* (Kirkness et al. 2010; Zhou et al. 2012). The protein sequences of ORs between insect orders also vary widely, except for that of Orco, a coreceptor necessary of OR functioning, that is highly conserved across insect orders (Larsson et al. 2004; Benton et al. 2006). Divergence in OR number and sequence is expected to have arisen from continuous evolutionary pressures, with each species presenting an OR repertoire that was adapted to its particular needs in a complex odor-filled world (Engsontia et al. 2014; Eyun et al. 2017; Robertson 2019). Functional characterization of OR repertoires in insects is an essential step toward understanding the mechanism of such adaptations, and more specifically to better understand plant–insect coevolution.

A large number of individual ORs have been functionally characterized in several species from different orders, but a global view of how a given species mobilizes a whole set of ORs has been detailed in very few species, namely *D. melanogaster*, the mosquito *Anopheles gambiae* (Carey et al. 2010; Wang et al. 2010), the ant *H. saltator* (Slone et al. 2017), and the moth *Spodoptera littoralis* (de Fouchier et al. 2017). These species are not related, nor are their ORs, which precludes fruitful comparative analyses. Thus, it is essential to accumulate more data on insect ORs for detailed inter- and intraorder comparisons to explore how this gene family evolved to fit with the chemical ecology of a given species (Robertson 2019). Lepidoptera represent a critical insect order for such an investigation, as it contains highly diverse organisms with over 160,000 described species that play extremely important ecological roles as plant pollinators and herbivores (Kawahara et al. 2019). However, only one OR repertoire has been characterized in Lepidoptera, that of the noctuid *S. littoralis* (de Fouchier et al. 2017), limiting intraorder comparative studies, especially in relation to plant interactions.

Here, we report the functional profiles of an unprecedented number of ORs in a Lepidoptera, the cotton bollworm *Helicoverpa armigera*, an important noctuid pest established as a model in chemical ecology studies because of its polyphagous diet, widespread distribution, and severely damaging impacts to agricultural systems (Jones et al. 2019). We reveal that the *H. armigera* OR (HarmOR) repertoire demonstrates a powerful ability to detect diverse plant volatiles. Comparing the HarmOR functional profiles with those of *S. littoralis* ORs, we reveal significant functional divergence in orthologous ORs, accompanied by a small set of functionally conserved OR pairs. Among those pairs, two orthologous ORs attracted our attention because of their remarkable and specific response to phenylacetaldehyde (PAA), a floral volatile characteristic of flowering plants that acts as an attractant to numerous species of Lepidoptera. Loss-of-function studies demonstrated the essential role of this OR in the detection of floral scent components in *H. armigera*. We next identified and functionally characterized orthologous ORs in numerous Lepidoptera species, revealing a conserved function in detecting PAA across Glossata.

## Results

### In Vitro Functional Screen Reveals the Robust Abilities of the HarmOR Repertoire for Sensing Many Plant Volatiles

In the *H. armigera* genome, 84 candidate OR genes have been annotated (Pearce et al. 2017) and previous transcriptome analyses identified at least 65 expressed ORs (Liu et al. 2012, 2014; Zhang et al. 2015; Guo et al. 2018), out of which 63 were expressed in the antennae of adults, including the coreceptor HarmOrco and seven pheromone receptors (PRs). The remaining 55 adult ORs were presumed to detect plant volatiles. We cloned 44 of these 55 ORs and heterologously expressed them in *Xenopus* oocytes for functional studies using two-voltage clamp electrophysiology. Each OR was challenged with a panel of 67 ecologically relevant plant volatiles (henceforth “odorants”). These 67 chemically diverse odorants (supplementary table S1, Supplementary Material online) were chosen based on their known effects on the physiology or behavior of *H. armigera* (or other moths) and were classified into three major chemical categories: terpenes, aromatics, and short-chain fatty acid derivatives (henceforth aliphatics).

**Fig. 1.**
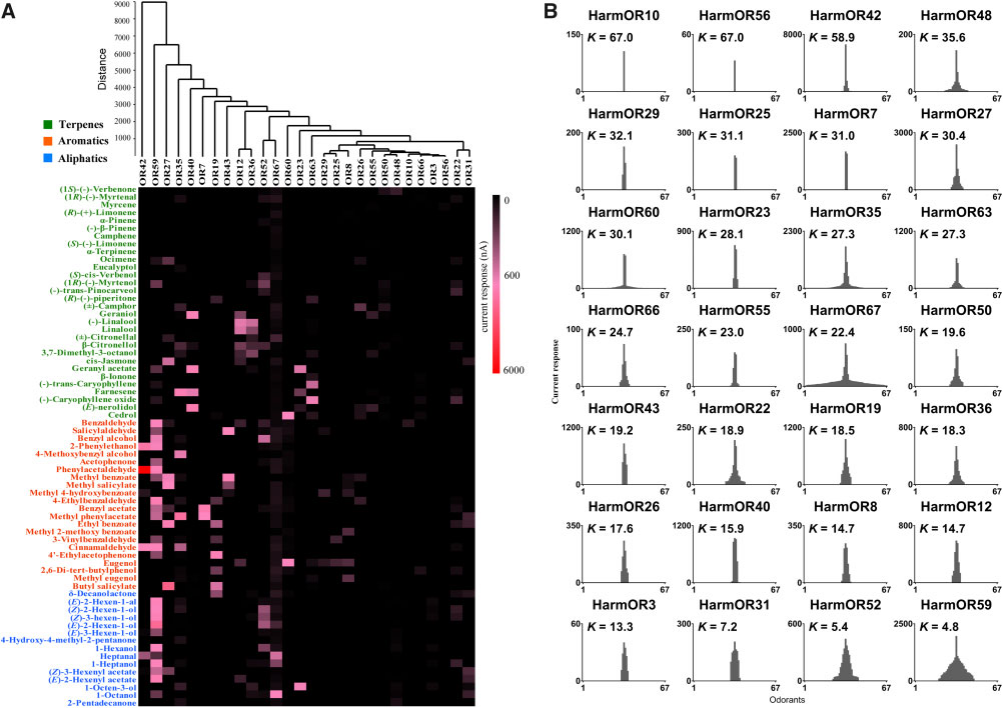
Responses of *Helicoverpa armigera* ORs (HarmORs) to plant volatiles by in vitro functional scanning. (*A*) The heat map was generated based on the mean current response of 28 functional HarmORs to 67 odorants at the dosage of 10^−4^ M. HarmORs are ordered based on the hierarchical cluster analysis of the mean responses. The current response was depicted by color intensity. The response values of each OR were acquired by testing four to eight oocytes. Three types of odorants are colored in orange (aromatics), blue (aliphatics), and green (terpenes). (*B*) Tuning breadth columns of 28 functional HarmORs to 67 odorants at the dosages of 10^−4^ M. Columns were generated by putting the largest value in the middle. Kurtosis values (*K*) were calculated to represent the degree of, where greater *K* values represent narrow tuning spectra and smaller ones represent broader spectra. The graphs were arranged by the *K* value of each HarmOR in descending order.

In preliminary screening, we used a high dosage (10^−4^ M) of odorants. At this concentration, a total of 28 ORs were found to be responsive to at least one odorant (fig. 1*A* and supplementary fig. S1, Supplementary Material online). Sixteen ORs did not exhibit any significant response to the tested odorants. Most functional ORs responded robustly to multiple odorants. The strongest response (up to ∼6,000 nanoampere, nA) we detected was that of HarmOR42 toward the aromatic compound PAA. Further, the response profiles of the 28 HarmORs appeared to be highly differentiated, with no apparent overlap in their tuning spectra (with the exception of HarmOR12 and 36) according to functional cluster analysis (fig. 1*A*).

Tuning spectrum of each HarmOR was analyzed, indicating ten HarmORs sensed structurally related chemicals (fig. 1*A* and supplementary fig. S1, Supplementary Material online): HarmOR3 responded to aliphatics; HarmOR7, 8, 10, 25, 29, and 43 to aromatics; and HarmOR40, 55, and 56 to terpenes. Four ORs (HarmOR31, 52, 59, and 67) were broadly tuned to multiple odorants belonging to the three chemical types. The other 14 HarmORs responded to more than one type of odorants. Tuning curves of each HarmOR were generated (fig. 1*B*), revealing a variety of tuning spectra, from very narrow to very broad with kurtosis values (*K*) ranging from 67.0 to 4.8. The ligands of the narrowly tuned HarmORs included a large number of important plant volatiles, such as benzaldehyde (the specific ligand of HarmOR10) and PAA (that induced the largest response of HarmOR42).

**Fig. 2.**
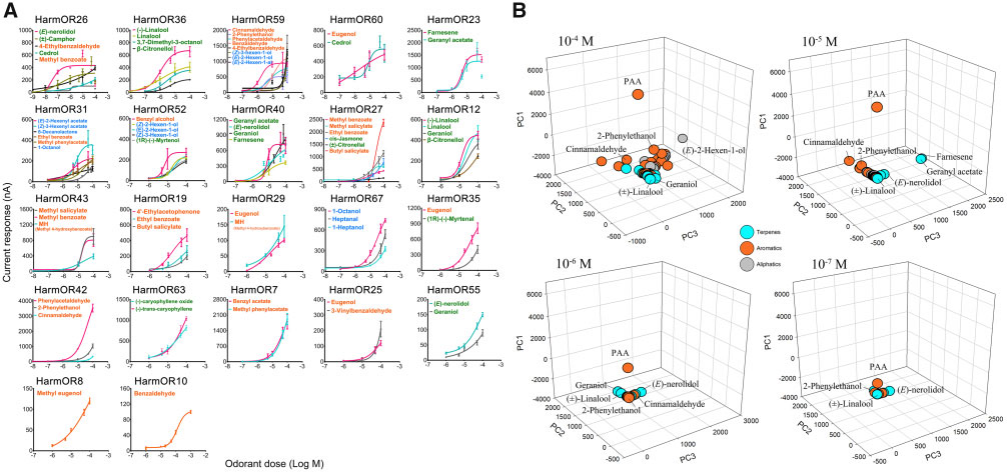
Dose–response profiles of the functional HarmORs toward their main ligands. (*A*) Dose–response curves of the functional HarmORs. The HarmOR repertoire was highly efficient in detecting terpenes and aromatics. Twenty-two functional HarmORs were arranged in an ascending order based on the EC_50_ value of the most sensitive ligand (listed in supplementary table S2, Supplementary Material online). Only a subset of the main ligands of HarmOR59 is shown. The most sensitive ligand(s) of each HarmOR are listed at the top and the font line color represents its chemical family (terpenes, green; aromatics, orange; aliphatics, blue). The current response values of each HarmOR to their ligands were acquired by testing four to eight oocytes. (*B*) Principal component analysis of current response values of the HarmOR repertoire to plant volatiles at different doses. The HarmOR repertoire was efficient for sensing certain terpenes and aromatics. In all graphs, vectors quantifying the responses of the 28 ORs to each odorant were projected onto a 3D region. These 3D representations capture 78.34% (10^−4^ M), 65.11% (10^−5^ M), 68.89% (10^−6^ M), 84.52% (10^−7^ M) of the variation in the original 28D data set across different odorant concentrations. Colors represent the different chemical classes: orange (aromatics), green (terpenes), and blue (aliphatics).

In order to expand our analysis on the response properties of HarmORs, we conducted dose–response analyses for the 28 functional HarmORs. For each HarmOR, ligands that were active at 10^−4^ M were tested at 10^−5^ M. Ligands that failed to trigger any response at 10^−5^ M were excluded from further analyses. Butyl salicylate, for instance, triggered HarmOR27 maximal response at 10^−4^ M dose but failed to evoke any current at 10^−5^ M dose. Six HarmORs (OR3, 22, 48, 50, 56, and 66) could not be activated by any ligand at 10^−5^ M. For the other 22 HarmORs, dose–response curves were generated and EC_50_ values of their major ligands were calculated (fig. 2*A* and supplementary table S2, Supplementary Material online). The HarmOR repertoire was highly efficient in terpene detection, since the lowest EC_50_ values were obtained for such compounds. Many of these terpenes have been proposed to be defensive chemicals of plants. For instance, the most sensitive response was obtained for the monoterpene (*E*)-nerolidol activating HarmOR26, with an EC_50_ of 2.89E-08 M. (*E*)-nerolidol is the direct synthetic precursor of DMNT and both compounds are major herbivore-induced compounds and act as defensive substances in many plants such as maize (Degenhardt and Gershenzon 2000; Turlings and Erb 2018). DMNT is also one of the oviposition deterring compounds found in larval frass of some Lepidoptera species such as *S. littoralis* (Anderson et al. 1993).

In addition to HarmOR26, we found that three other HarmORs (OR23, 36, and 40) were very sensitive to terpenes such as linalool and farnesene, other major herbivore-induced compounds, with EC_50_ values lower than 10^−5^ M. The HarmOR repertoire was also efficient in detecting aromatics, with a large array of 14 ORs being the most sensitive to aromatic compounds, even though their sensitivities were lower than that of ORs tuned to terpenes. For comparison, only seven and two ORs were most sensitively tuned to terpenes and aliphatics, respectively. Notably, four HarmORs were tuned to eugenol as their most sensitive ligand.

We then performed principal component analysis (PCA) of the 67 odorants across the 28 ORs in different dosages (10^−4^ to 10^−7^ M) (fig. 2*B*), which further indicated that HarmOR responses were odorant structure- and concentration-dependent and that the HarmOR repertoire was efficient for sensing certain terpenes and aromatics. Vectors quantifying the responses of the 28 ORs to each odorant were projected onto a 3D landscape. This 3D representation captured 78.34% (10^−4^ M), 65.11% (10^−5^ M), 68.89% (10^−6^ M), and 84.52% (10^−7^ M) of the variation in the original 28D data set across different odorant concentrations. In most cases, odorants that share common structural features clustered together by chemical groups, indicating that odorant position in odor space was largely, but not exclusively, dependent on their chemical structure. In addition, the capacity of the *H. armigera* odor space was highly dependent on odorant concentration. As the concentrations of odorants decreased, their positions in the odor space converged, with the ability to discriminate different odorants diminishing. Remarkably, at the very low odorant dosage concentration of 10^−7^ M, the HarmOR repertoire still responded to terpenes including (*E*)-nerolidol, linalool, and aromatics including PAA, 2-phenylethanol, which are very common volatiles emitted by angiosperms.

### Substantial Functional Differentiation of Orthologous ORs between *H. armigera* and *S. littoralis*

**Fig. 3.**
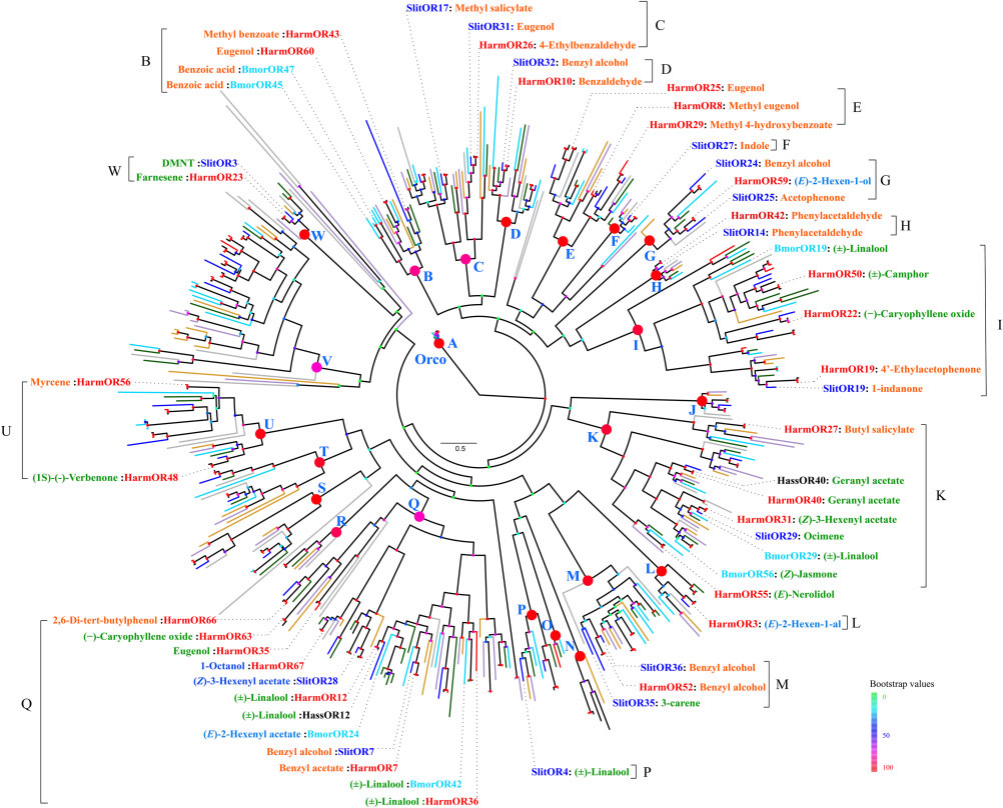
Phylogenetic analysis of ORs from Lepidoptera. The phylogenetic tree was built using the amino acid sequences of 461 ORs from eight species based on the maximum likelihood algorithm. Twenty-three major clades with high node support (bootstrap values >80) were labeled with red or pink dots and blue letters (A–W). The main ligand of each functional HarmORs as well as other reported data were arranged around the phylogenetic tree, and is colored using the same color code as in figure 1 according to its chemical family. Each branch of the OR gene tree was colored differently according to species: red (*Helicoverpa armigera*, Harm), dark blue (*Spodoptera littoralis*, Slit), black (*Helicoverpa assulta*, Hass), sky blue (*Bombyx mori*, Bmor), green (*Manduca sexta*, Msex), brown (*Ostrinia furnacalis*, Ofur), violet (*Chilo suppressalis*, Csup), and gray (*Grapholitha molesta*, Gmol). Similarly, the functional identified ORs were colored using the same scheme.

In recent years, a large number of OR sequences have been identified in many lepidopteran species, and the functions of some ORs have been clarified. The abundant functional data generated in our study allowed us to re-examine the evolution of lepidopteran ORs in light of their sensing functions. As an initial step, we built a phylogenetic tree of lepidopteran ORs based on 461 ORs from eight lepidopteran species. These ORs clustered into 23 major clades (A–W) with high support by bootstrap values (fig. 3). The tree was rooted at clade A (the so-called “Orco” clade), in which the receptors are known to be highly conserved among a wide range of insect species. The 65 expressed HarmORs were scattered over the 23 clades, illustrating the diversity of ORs within a given Lepidoptera species. No apparent species-specific OR clade were observed and most ORs have orthologs in other species. The PR clade (clade V) was easily distinguishable, as all the ORs in this clade are PRs from different lepidopteran species, including seven PRs from *H. armigera*. Some clades consisted of a limited number of ORs from each species, all close together (such as clades H, J). Other clades expanded widely (*e.g.*, clades I, K, M, Q, and V), which is consistent with the rapid evolution of insect ORs.

The phylogenetic tree was decorated with functional data outlining the main ligand of each OR based on their tuning spectrum (when available). By comparing the major ligands of ORs from reported species, we found substantial degree of functional differentiation within most clades, which is consistent with the rapid expansion of ORs within insects (Hansson and Stensmyr 2011; Engsontia et al. 2014; Robertson 2019). For instance, some orthologs such as HarmOR42–SlitOR14 or HarmOR36–BmorOR42, shared the same main ligand. Reversely, some receptors that belong to different clades take the same compound as their main ligand. For example, both HarmOR25 from clade E and HarmOR60 from clade B have the same main ligand eugenol.

**Fig. 4.**
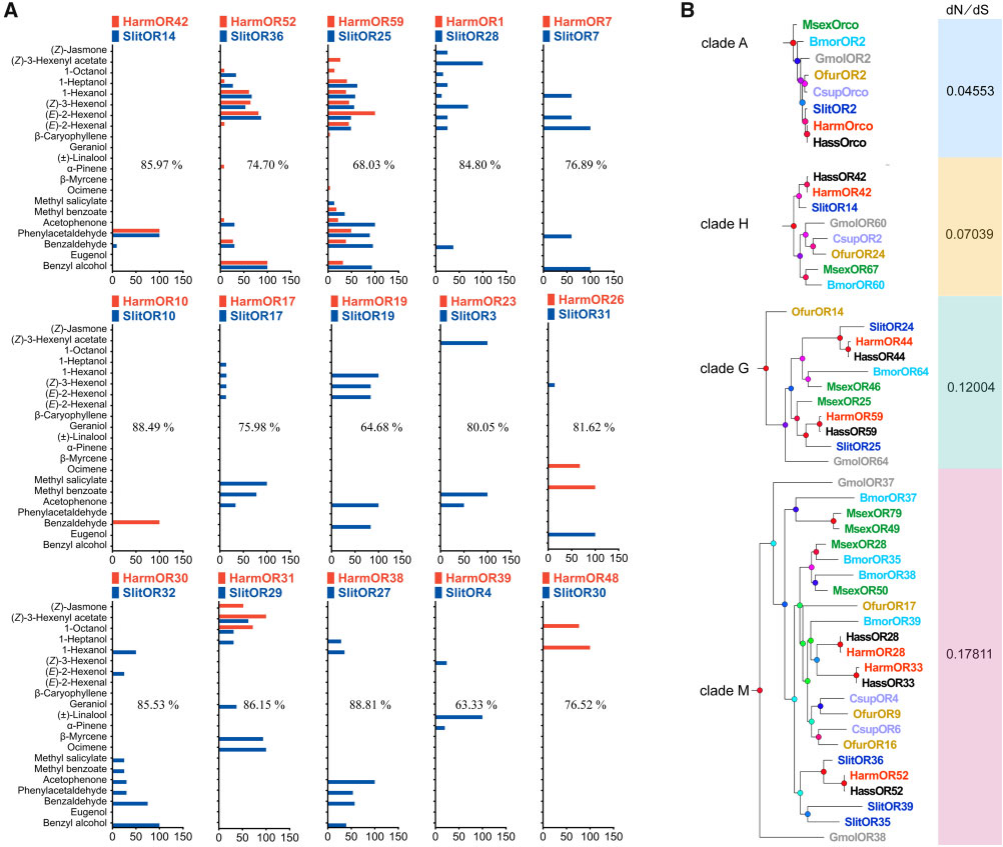
Functional differentiation and conservation of orthologous ORs between *Helicoverpa armigera* and *Spodoptera littoralis*. (*A*) Tuning spectra comparison between *H. armigera* (red) and *S. littoralis* (blue). The responses of fifteen orthologous OR pairs against 21 overlapping odorants are shown. Twelve of the 15 pairs that share high sequence identities (from 63.33% to 88.49%) present nonoverlapping response spectra. The first three pairs of orthologous ORs showed identical response spectra. The mean response values of each OR were normalized by defining the maximal response as 100. The amino acid sequence identity of each orthologous OR pair is shown in each diagram. (*B*) Selection pressure analysis on four orthologous ORs clades (A, Orco; H, OR42; G, OR59; M, OR52) in the phylogenetic tree of figure 3. The d*N*/d*S* ratios of clades A, H, G, and M are listed behind the branches. The value with d*N*/d*S* <1 indicates the genes within one clade have evolved under purifying selection.

We conducted a deeper comparison of the whole response spectrum between *H. armigera* and *S. littoralis* orthologous ORs, as large arrays of ORs have now been characterized in both species (de Fouchier et al. 2017 and the present study). Based on the constructed phylogenetic tree (fig. 3), we identified 15 pairs of orthologous ORs that have been functionally characterized in the two species. We compared the response spectra of these OR pairs using the mean normalized response value to 21 overlapping odorants used in both studies (fig. 4*A*). This revealed a large degree of functional differentiation among orthologous ORs, with twelve of the 15 pairs presenting nonoverlapping response spectra, although they share high sequence identities (from 63.33% to 88.49%).

In contrast, three pairs of orthologous ORs showed identical response spectra, despite having sequence identity values within the same range as that of the aforementioned 15 functionally divergent pairs. Among these three pairs, the most highly conserved pair consisted of HarmOR42–SlitOR14 (sequence identity = 85.97%), with both responding with high specificity and sensitivity to the same ligand: PAA. HarmOR52–SlitOR36 (identity = 74.70%) and HarmOR59–SlitOR25 (identity = 68.03%) were broadly tuned with similar spectra for sensing several aromatics and aliphatics, including PAA, benzaldehyde, and (*Z*)-3-hexenol. All ligands for these functionally conserved ORs have been reported as common volatiles from known host plant species. The aromatics PAA and benzaldehyde are two of the most common floral volatile compounds, as revealed by a large-scale study of plant families at different taxonomic levels (Knudsen et al. 2006; Schiestl 2010). Generally, our comparison of the tuning spectra of orthologous ORs indicated substantial functional differentiation between *H. armigera* and *S. littoralis* ORs, but also clearly highlighted that some ORs have retained the same functions, suggesting that the ligands of such ORs may represent particularly impactful fitness-related cues.

We further investigated the HarmOR42 and SlitOR14 pair, as both ORs had a very robust and specific response to PAA. In the phylogenetic analysis, they defined a unique clade, with one representative OR from all species, and their especially short branch lengths indicated relatively small genetic distance between each clade member (fig. 4*B* and fig. 3—clade H). We therefore conducted a selection pressure analysis on each clade (supplementary table S3, Supplementary Material online; fig. 4*B*), which suggested that all OR clades, including the HarmOR42 and the Orco clades, have evolved under strong purifying selection. This is consistent with previous studies on insect OR evolution (Nei et al. 2008; Missbach et al. 2014; Yang et al. 2017; Robertson 2019). In view of this strong selective pressure and of the observed conserved functions for PAA detection in *H. armigera* and *S. littoralis*, we hypothesized that the lepidopteran HarmOR42 orthologs likely function in flower sensing.

### A Conserved or Lineage in the Glossata Suborder for Sensing a Common Indicator of Flowering Plants

**Fig. 5.**
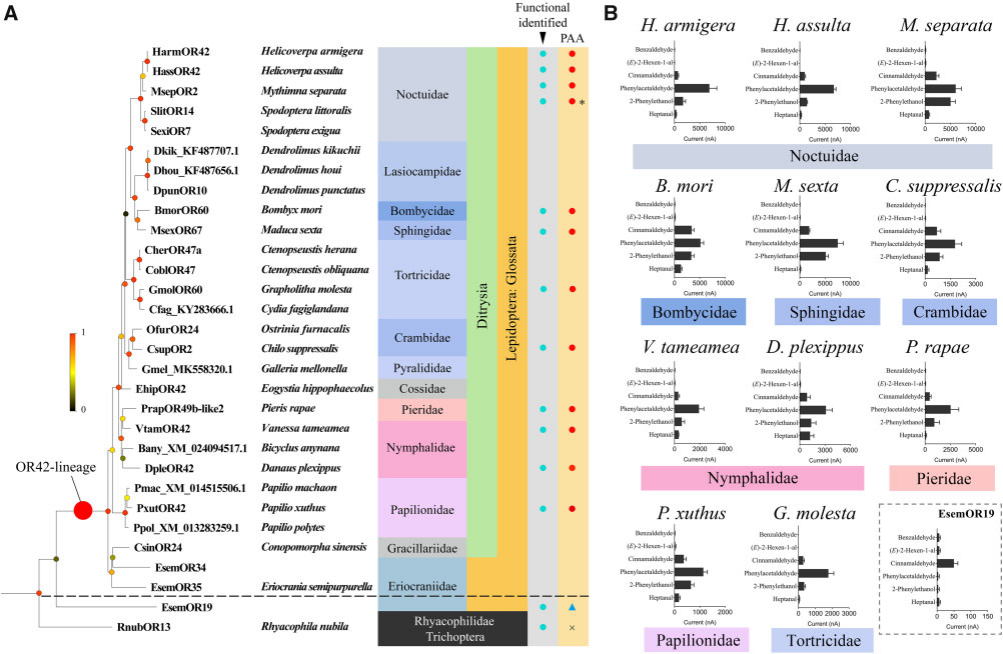
Unique lineage of OR42 orthologs across moths and butterflies. (*A*) Orthologous ORs of HarmOR42 in Lepidoptera species constitute a unique lineage with high node support and the functional identified OR42 orthologs in twelve species respond to PAA. The OR42 clade was picked from a phylogenetic tree of 1,619 ORs from 31 species belonging to the Lepidoptera (30 species from 13 families) and the Trichoptera (1 species) orders. The color dots on the nodes indicate bootstrap support values. Ditrysia and Glossata crown groups are highlighted in green and yellow. Functionally characterized orthologous ORs are marked by cyan dots, and those tuned to PAA (phenylacetaldehyde) are marked by red dots. A blue triangle behind EsemOR19 represents its lack of response for PAA, but responsivity for another ligand. The cross for RnubOR13 denotes that this OR is not activated by any of the tested ligands. The asterisk indicates that the function of SlitOR14 for sensing PAA was reported by de Fouchier et al. (2017). (*B*) Tuning spectra of OR42 orthologs from 11 Lepidoptera species, and one homolog (EsemOR19) from outside the OR42-lineage. All the orthologs in the 11 Lepidoptera species have similar response profiles, which robustly tuned to PAA. The tuning spectra of the 11 orthologs completely overlap based on a screen of six compounds at 10^−4^ M. EsemOR19 tuned to cinnamaldehyde, with a very weak current. Families are indicated beneath each histogram. The current response value of each OR42 ortholog was acquired by testing four to eight oocytes.

To test our hypothesis about lepidopteran HarmOR42 orthologs as major flower sensing receptors, we first searched for candidate HarmOR42 homologs among 1,619 ORs from 31 species, including 30 Lepidoptera and one Trichoptera (*Rhyacophila nubila*) by constructing a new phylogeny. We identified homologs exclusively in the Lepidoptera. They constitute a unique lineage with high node support (fig. 5*A*), with representatives from almost all of the investigated Lepidoptera species (27 out of 30). These 27 species belong to 13 families of butterflies and moths, including the non-ditrysian moth *Eriocrania semipurpurella*, which belongs to a basal lineage of the Glossata suborder (Kawahara et al. 2019).

We found only one HarmOR42 ortholog in each species, with the exception of *E. semipurpurella*, for which two partial nonoverlapping sequences were detected (EsemOR34 and EsemOR35) (Yuvaraj et al. 2017). However, alignment with the other HarmOR42 orthologs suggested that these two *E. semipurpurella* fragments might be in fact two parts of a single OR, as they respectively overlap with different part of other orthologs with high amino acid identities (supplementary fig. S2, Supplementary Material online). Thus, it appears that this OR lineage has not expanded since its emergence in Glossata. Further, sequences of the HarmOR42 orthologs shared multiple conserved domains and had an average of 67% amino acid identity, suggesting a conserved function; note that the sequences of several orthologs were partial (supplementary fig. S2, Supplementary Material online). Taking together, these results support that the ancestral ortholog of HarmOR42 evolved from a non-ditrysian species at the base of the Glossata lineage and that the orthologs have retained a conserved possible PAA-sensing function across Glossata species.

We gathered additional experimental evidence to support this conservation of a PAA-sensing function by performing in vitro functional characterization of HarmOR42 orthologs from 11 lepidopteran species belonging to eight families (fig. 5*B*). All of the HarmOR42 orthologs exhibited similar response profiles, with each robustly tuned to PAA as its best ligand. It should be noted that the species studied here included three Noctuidae species (*H. armigera*, *H. assulta*, and *Mythimna separata*), four moths from Bombycidae (*Bombyx mori*), Crambidae (*Chilo suppressalis*), Sphingidae (*Manduca sexta*), and Tortricidae (*Grapholitha molesta*) (Mitter et al. 2017; Kawahara et al. 2019), and four diurnal butterfly species from diverse families (*Vanessa tameamea*, *Papilio xuthus*, *Danaus plexippus*, and *Pieris rapae*) (fig. 5*B*).

We also examined the response profile of EsemOR19 from *E. semipurpurella*, an OR that falls close to the HarmOR42-defined clade but that is not considered as a HarmOR42 ortholog. EsemOR19 showed no response to PAA, but responded to cinnamaldehyde, one of the minor ligands for all of the HarmOR42-orthologs. We also characterized the function of RnubOR13 from *R. nubila* (Trichoptera), a species from a sister order of Lepidoptera. This OR was activated neither by PAA nor by any of the compounds in the test panel. Unfortunately, the putative *E. semipurpurella* OR that would consist of EsemOR34 + 35 and that falls at the base of the HarmOR42 clade could not be tested as it is incomplete. Collectively, these results strongly suggest that the function of HarmOR42-orthologs for sensing PAA is conserved across diverse moths and butterflies.

### HarmOR42 Is Essential for Floral Scent Sensing in *H. armigera*

**Fig. 6.**
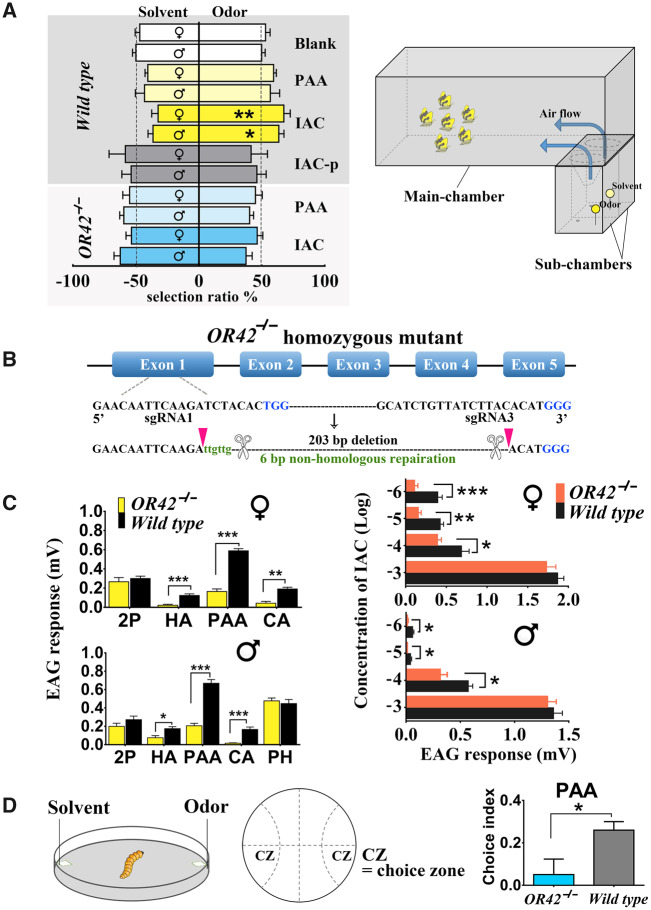
In vivo functional studies indicating the key role of HarmOR42 in sensing floral scent and host plant cues. (*A*) (Left) Behavioral responses of wild-type and HarmOR42-lacking mutants of *Helicoverpa armigera* moths to odorant or blends in two-choice olfactometers. Both of the female and male wild-type moths were significantly attracted by the floral scent mixture IAC that mainly contain PAA. The mutants lost their preference to IAC compared with wild type. PAA, phenylacetaldehyde; IAC blend (50% PAA, 20% salicylaldehyde, 10% methyl 2-methoxybenzoate, 10% linalool, and 10% (*R*)-(+)-limonene); IAC-p, IAC blend minus PAA; Blank, solvent. Asterisks in black font represent the statistical differences in distribution compared with solvent blank groups (*0.01 < *P *<* *0.05; **P <* *0.01). Four to six repetitions were performed for each chemical or blend. Thirty moths (either males or females) were tested in each repetition. (Right) Schematic representation of the two-choice olfactometer. (*B*) CRIPSPR/Cas9-based knock-out of *HarmOR42* gene in *H. armigera*. Target sequences of the two sgRNAs (black) and the PAM sequences (blue) are shown under the exon cluster. The obtained mutation consisted of a 203-bp nucleotide fragment deletion and a 6-bp nonhomologous insertion (green) in the genome. (*C*) EAG responses of wild-type and HarmOR42 mutant moths to four major ligands (1 μg) of HarmOR42 (Left) and different dosages of the blend IAC (Right). The EAG response of HarmOR42 knock-out mutants to PAA or IAC was significantly decreased compared with wild-type moths. 2P, 2-phenylethanol; HA, heptanal; PAA, phenylacetaldehyde; CA, cinnamaldehyde; PH, Z11-16: Ald. Fifteen repetitions were performed for each odorant or blend. Asterisks represent the statistical difference by Student’s *t*-test (*0.01 < *P *<* *0.05, **0.001 < *P *<* *0.01, ***P <* *0.001). (*D*) Petri dish behavioral experiments of *H. armigera* larvae to PAA. The larvae of wild-type *H. armigera* were strongly attracted by PAA. The HarmOR42 knock-out mutants lost their preference to PAA significantly. Ten repetitions were performed for either wild type or mutants. Ten third instar larvae were used in each repetition. Asterisks represent the statistical difference by Student’s *t*-test with both strains (*P <* *0.05).

As all the HarmOR42 orthologs in Glossata exhibited a conserved function in detecting PAA, the most common volatile compound emitted by flowers and host plants, we further investigated if these ORs are essential in trigerring the behavior to PAA in vivo. In a first step, we tested the attractiveness of PAA and a commercial mix of floral attractants, which takes PAA as its principal component (IAC) (Cork 2016; Whitfield et al. 2019), to *H. armigera* moths using a two-choice behavioral assay in a choice box (fig. 6*A*). PAA was more attractive to *H. armigera* moths than the solvent control, but the results were not statistically significant. The floral attractant mix IAC elicited a significant positive response in both females and males, but when PAA was removed from IAC, the incomplete IAC blend (IAC-p) was not attractive anymore to *H. armigera*. These results confirm the strong attractiveness of IAC to *H. armigera* moths and demonstrate the essential role of PAA for the mix activity.

We next conducted a loss-of-function study by generating a HarmOR42-lacking *H. armigera* strain by CRISPR/Cas9 targeting the first coding exon. Homozygous mutants were obtained with a large segment deletion of 203 bp and a 6-bp nonhomologous insertion in the genome (fig. 6*B*), introducing a premature stop codon in the coding sequence. Electroantennography (EAG) experiments showed that the response of HarmOR42 knock-out mutants to PAA was significantly decreased in both females and males compared with wild-type moths (fig. 6*C*), indicating that HarmOR42 greatly contributed to the sensing of PAA. Similarly, the response to cinnamaldehyde and heptanal, which were two of the minor ligands of HarmOR42, was also significantly decreased. There was no significant difference of EAG responses to 2-phenylethanol, another minor ligand of HarmOR42, between the mutant and wild-type moths. The most likely hypothesis is that HarmOR42 is not the major receptor for sensing this compound in *H. armigera*. As a control, we tested the response of male moths to the sex pheromone component Z11-16: Ald that is not a ligand of HarmOR42. As expected, we observed no difference between the EAG responses of mutant and wild-type moths to this compound.

Further, we registered the EAG responses to the floral attractant IAC blend at different doses. The results again showed a significant decrease in the response of mutant moths compared with wild type, except at the highest dose (10^3^ times dilution) (fig. 6*C*). These results indicated that HarmOR42 greatly contributes to the antennal response to PAA. To test whether HarmOR42 could also be essential in the moth behavioral attraction to PAA and IAC, we further challenged HarmOR42 knock-out mutants in the choice assay. We found that both male and female mutants lost their preference to IAC compared with wild type (fig. 6*A*), which strongly confirmed the essential role of HarmOR42 in sensing floral scent.

As previous studies revealed that *HarmOR42* is also expressed in the larval stage of *H. armigera* (Di et al. 2017), we tested the attractive effect of PAA on third instar larvae in a simple Petri dish assay (fig. 6*D*) and revealed that PAA significantly attracted larvae. The HarmOR42 knock-out mutant larvae, as expected, lost their preference to PAA. Our results provide strong evidence that PAA, via its detection by HarmOR42, is an important host plant cue in both larval and adult stages of *H. armigera*.

## Discussion

Functional characterization of a large OR repertoire is crucial to understand how a given species uses olfactory cues to meet its ecological needs. Such characterization is particularly relevant in herbivorous Lepidoptera, an insect order that contains multiple species with diverse ecological requirements that can severely damage a variety of agricultural ecosystems. Yet, such a large functional effort has been conducted in only one Lepidoptera species, the polyphagous noctuid *S. littoralis*, precluding comparative studies across the order. Here, we have characterized the response profiles of an unprecedented number of ORs in another noctuid, the prominent pest *H. armigera*, to a broad panel of diverse plant volatiles. With a success rate of 63.6%, 28 out of 44 HarmORs could be activated by at least one odorant, which is close to the ratio obtained in the previous *A. gambiae* OR functional study (62.5%, 45 out of 72 ORs) using the same expression system (Wang et al. 2010), as well as in studies using OR expression in *Drosophila* neurons (Carey et al. 2010, 69% success rate; de Fouchier et al. 2017, 68% success rate). The remaining 17 HarmORs failed to respond to any odorants on the panel. A possible reason might be that these OR ligands do not belong to the odorant panel that we tested here, or that they did not express correctly in the oocyte membrane, as suggested in these previous studies.

For the functional ORs, we found large difference in their respective sensitivity toward ligands (from 30 to 6,000 nA), as observed for other species using the same expression system (Wang et al. 2010). Some ORs showed modest response to any odorants, even when these have been tested at a very high concentration. Observation of modestly tuned ORs have been reported in other insect species using the same expression system as in our study (Wang et al. 2010), but also using in vivo functional expression in *Drosophila* neurons (the so-called empty neuron system or the *Orco-Gal4* system) (de Fouchier et al. 2017; Slone et al. 2017). As well, large but more modest variations in response amplitude between ORs have been reported in previous studies using expression in *Drosophila* neurons. It has to be noticed that variation in response amplitudes are not comparable between the two expression systems. Whereas oocyte responses (measured as injected current for voltage clamp) can vary from 0 to 6,000 nA (Wang et al. 2010, this study), *Drosophila* neuron responses usually do not exceed 250 spikes/s, because neuron spiking activity is physiologically limited (Carey et al. 2010; de Fouchier et al. 2017; Slone et al. 2017). Several hypotheses can be proposed to explain such differences in OR sensitivity (whatever the expression system). First, it is possible that the main(s) ligand(s) (those inducing high responses) of modestly tuned ORs are not present in the tested panel. Second, is also possible that protein expression level varies according to ORs, depending if they are correctly addressed to the membrane of oocytes/*Drosophila* neurons (depending on chaperon proteins/Orco). Last, differences in sensitivity may represent real/endogenous properties of ORs in vivo. Such differential sensitivity may indeed participate in concentration coding, with some ORs being activated at low doses, others being activated at higher doses (de Fouchier et al. 2015).

The functional characterization of a large array of HarmORs suggests that the peripheral olfactory system of adult *H. armigera* possesses powerful capabilities for sensing and distinguishing plant-volatile cues. The HarmOR repertoire exhibited a strong ability for detecting aromatics and terpenes, which are among the most common constituents of plant odors emitted by both flowers and leaves, endowing *H. armigera* to efficiently orientate to host plants. We found that the tuning spectrum widths of ORs diverged largely. Some generalist ORs, such as HarmOR52 and OR59, were broadly tuned to multiple host plant volatiles belonging to different chemical classes. Such broadly tuned ORs have been identified in other insects (Carey et al. 2010), including Lepidoptera (de Fouchier et al. 2017). Their large tuning, together with their various sensitivity toward common ligands (see Discussion upper), suggest that they participate in peripheral combinatorial coding of a large panel of odorants as well as in encoding variations in odorant quantity (de Fouchier et al. 2017). We also found evidence of some specialist HarmORs, tuned to one or a few structurally similar compounds even in high concentration. Such narrowly tuned insect ORs are usually associated with vital behaviors and have been described in *D. melanogaster, A. gambiae*, and *S. littoralis* (Hallem and Carlson 2006; Wang et al. 2010; de Fouchier et al. 2017). They usually participate in labeled line olfactory circuits employed by insects for sensing pheromone or ecologically chemicals cues (Haverkamp et al. 2018). Among examples of insect ORs involved in labeled line circuits, one can cite the *Drosophila* pheromone receptor DmelOR67d detecting male-produced pheromone 11-*cis*-vaccenyl acetate (Kurtovic et al. 2007) and the *M. sexta* MsexOR1 sensing pheromone component bombykal, which alone elicits male attraction by activating specific peripheral olfactory neurons (Wicher et al. 2017). Another example of such labeled line is the geosmin circuit in *Drosophila*. Flies use a single class of olfactory neurons expressing exclusively DmelOR56a to detect geosmin, a key compound of harmful bacteria and mold that elicits innate avoidance behavior of flies (Stensmyr et al. 2012). The identification of narrowly tuned HarmORs suggests that they may be involved in such labeled line circuits. One example could consist of HarmOR42 for sensing PAA, because it is reported to exclusively mediate attraction in both sexes of *H. armigera* and its knock-out resulted in losing preference to PAA in both larvae and adults of *H. armigera*.

Combining phylogenetic and functional analysis of ORs from eight lepidopteran species, we found on the one hand great functional differentiation of ORs within and among clades, and on the other hand some functional conserved orthologs between species. Another striking point is that some Lepidoptera ORs shared the same main ligands although they were located in distantly related phylogenetic clades. For instance, we found that HarmOR25 in clade E and HarmOR60 in clade B shared eugenol as their main ligand. Previous works on other Lepidoptera (de Fouchier et al. 2017) and Diptera (Hallem and Carlson 2006; Wang et al. 2010) species also reported that a given odorant can be detected by divergent ORs, whatever the expression system used (oocytes or *Drosophila* neurons). This phenomenon is observed within the same species (*H. armigera* as cited upper, but also in *S. littoralis*, de Fouchier et al. 2017) and between species (for instance, the *B. mori* linalool receptor appeared to be distantly related to the *S. littoralis* linalool receptor in de Fouchier et al. [2017] analyses). We cannot exclude that what appeared as the main ligand for a given OR is in fact not its main ligand, because of limited odorant panels and use of sometimes high doses of odorants. However, most studies—including our—conducted dose–response analyses, revealing that ORs recognizing the same ligand usually have different detection thresholds. Thus, it is likely that OR functional redundancy together with different sensitivity are at the core of combinatorial coding of odorant and concentration detection.

Deeper analyses can be done when odorant panels used in different studies are overlapping, which is the case between our study and the previous one in *S. littoralis* (de Fouchier et al. 2017). By comparing the response spectra of orthologous ORs in *H. armigera* and *S. littoralis*, we revealed substantial functional differentiation, although both species are polyphagous with largely overlapping host plants, which is consistent with the rapid evolution of insect ORs (Robertson 2019). These findings indicate that there is no correlation between sequence similarity and functional property of ORs, as already observed: ORs with relative high sequence identities often exhibit different functional property, and single point mutations can alter one OR function (Mitsuno et al. 2008; Leary et al. 2012; Cao et al. 2016; Yang et al. 2017; Auer et al. 2020). We also identified pairs of orthologous ORs with functional conservation between the two species. These structurally and functionally conserved ORs were tuned to common, essential host plant volatiles, among which was PAA. The occurrence of functionally conserved and divergent orthologous OR pairs in the two herbivorous noctuids provides new insights into the evolution of ORs among Lepidoptera species, suggesting two evolutionary pathways. One pathway would favor functional diversification to allow exploration or adaptation to new environments; the other would maintain functions for basic survival and reproduction.

Notably, we revealed that the PAA-functionally conserved OR lineage extended across Glossata, which contains up to 99% of lepidopteran species (Kawahara et al. 2019). Based on available genome and transcriptome data, we identified a unique HarmOR42 orthologous OR in almost all the investigated Glossata species, spanning 13 families and nine superfamilies. These species are derived from several taxonomically and ecologically diverse Ditrysia groups such as Noctuoidea, Pyraloidea, and Papilionoidea (Roe et al. 2009; Mitter et al. 2017; Kawahara et al. 2019) and exhibit dramatic host plant, diet regime, and habit diversity, from host specialists such as the monarch butterfly *D. plexippus*, a diurnal butterfly feeding on milkweed, to highly polyphagous nocturnal species such as *H. armigera*. All of the HarmOR42 orthologous genes were found as single-copy genes in each species and were under purifying selective pressure, as were other important genes such as Orco, suggesting an essential role for HarmOR42 across species.

Further functional characterization of the HarmOR42 orthologs from 11 moth and butterfly species revealed a conserved function, as the orthologs had completely overlapping tuning spectra with PAA being the main ligand for activation. PAA is a universal compound emitted by a large number of flowering plants across different taxonomic levels. Furthermore, PAA is among the unique compounds emitted by angiosperms compared with gymnosperms, and it is supposed to be a good indicator of nectar sources as the amount of PAA is tightly associated with nectar sugar and pollen amounts (Schiestl 2010; Knauer and Schiestl 2015). PAA is also released by many important crops such as maize, cotton, tomato, and is a major contributor to the flavor of tomato fruits (Tieman et al. 2006). Multiple behavioral studies have demonstrated the broad attractiveness of PAA (usually as a major component in odor blends) to various Lepidoptera species, especially to many diurnal butterflies and agricultural pests. Thus, PAA stands out from complex mixtures of plant volatiles and maintains its essential role as a food indicator in the long coevolutionary history between Lepidoptera insects and angiosperms. Our results indicate that PAA detection is ensured by the HarmOR42 lineage that evolved under purifying selection, and that HarmOR42 maintained its function during the evolution of Glossata over approximately 240 million years. The crucial role of HarmOR42-orthologs in detecting PAA has been verified in *H. armigera* through loss-of-function studies and we can speculate that it defines the common molecular basis of the attractiveness of PAA in moths and butterflies.

The most ancestral ortholog of HarmOR42, although with a partial sequence (EsemOR34 and EsemOR35), was detected in the non-Ditrysia species *E. semipurpurella* (Eriocranioidea) that belongs to the basal Glossata clade in Lepidoptera. Due to the high sequence similarity among the two fragments and other HarmOR42-orthologs (supplementary fig. S2, Supplementary Material online), we anticipate this OR in *E. semipurpurella* is likely to function in detecting PAA, although this remains to be verified. We did not detect any OR42 orthologs beyond Glossata, but because of the paucity of genomic data on insect species outside Glossata, it is difficult to clearly assess if HarmOR42 orthologs are restricted to Glossata. Within Glossata, we did not identify any EsemOR19 ortholog (EsemOR19 from *E. semipurpurella* was close to the OR42-lineage and responded to a structural analog of PAA, but not to PAA). Clearly, more OR sequences and functional data are needed to propose a scenario on PAA-sensing OR evolution in Glossata, but we can presume that the functional ancestor of HarmOR42 evolved at least in basal Glossata species. As it is well established that insect ORs evolve through gene gain-and-loss, and considering that EsemOR19 is at the base of the HarmOR42 clade, a hypothetical scenario is that the *EsemOR19* gene duplicates, one copy (EsemOR34–35) gaining function toward PAA sensing, and the other copy being lost in Glossata. Genomic data for *E. semipurpurella* are needed to check if EsemOR19 and EsemOR34–35 are indeed in tandem on the same chromosome and with similar intron–exon organization, which would argue in favor of a gene duplication event.

It is speculated that adults of Eriocranioidea have the precursory behavior of nectar feeding using their proboscis, and the time when the common ancestor of nectar-feeding Lepidoptera first appeared overlapped with the estimated period when flowering plant crown groups vastly expanded and diversified (Kawahara et al. 2019). A possible evolutionary scenario might be that the common ancestor of Glossata coevolved a tube-like proboscis—which endows the ability to collect nectar from flowering plants—and the necessary receptors for detection of key floral scent compounds including PAA, an aromatics compound exclusively reported in angiosperm (Schiestl 2010), make foraging process more efficient. Our result thus sheds light on the mechanisms of ecological adaptations of Glossata species with angiosperms and also defines a potential target for behavioral regulation of a wide range of Lepidoptera pest species.

## Materials and Methods

### Insect Rearing

*Helicoverpa armigera* were reared at the Institute of Plant Protection of the Chinese Academy of Agricultural Sciences in Beijing, China. The conditions for insect rearing were: 16:8 h (light:dark) photoperiod at 27 ± 1 °C and 65 ± 5% relative humidity. Larvae were reared on an artificial diet. Pupae were separated according to sex, and males and females were placed in separate glass tubes. Adults were fed on 10% (w/v) sucrose water after emergence each day until they were used for experiments.

### In Vitro Functional Characterization of ORs

Full-length coding sequences of 45 ORs (including Orco) of *H. armigera*, and OR42 and Orco orthologs in *B. mori*, *H. assulta*, *G. molesta*, and *M. separate* were amplified from the antennal cDNA of each species adults by PCR with specific primer pairs of each gene. For *C. suppressalis*, *M. sexta*, *E. semipurpurella, V. tameamea, P. xuthus*, *D. plexippus*, and *P. rapae*, the full-length gene of OR42 and Orco orthologs were synthesized (Sangon Biotech, Shanghai, China) according to reported data. The gene accession number of each OR and the full-length cloning primers of the genes are listed in supplementary table S4, Supplementary Material online.

Functional characterization of individual ORs was performed by heterologous expression in *Xenopus* oocytes combined with a two-electrode voltage-clamp system (Wang et al. 2010). Briefly, the full-length gene of each OR and Orco were subcloned into the eukaryotic expression vector pT7TS. cRNAs were generated from linearized expression vectors using the mMESSAGE mMACHINE T7 kit (Ambion, Austin, TX). Then, the cRNA mixture of ORx and Orco (27.6 ng each) was injected (Nanoliter 2010, WPI Inc., Sarasota, FL) into oocytes (stage V–VII). The respective Orcos of different species were used for functional studies of OR42 orthologs except for the four butterfly species (*V. tameamea, P. xuthus*, *D. plexippus*, and *P. rapae*), in which we used the Orco from *P. xuthus*. After incubation in nutrient solution at 18 °C for 3–5 days, the response profile of each oocyte to multiple plant odorants was recorded via a two-electrode voltage clamp (OC-725C oocyte clamp, Warner Instruments, Hamden, CT) at a holding potential of −80 mV. Data were acquired by using a Digidata 1440 A and were analyzed by pCLAMP 10.2 software (Axon Instruments Inc., Union City, CA).

Stock solutions of each odorant were prepared at 1 M using DMSO as a solvent, and each odorant was diluted in 1× Ringer’s solution to the indicated concentrations for electrophysiological recording. For functional screening of each HarmOR, a panel of 67 odorants belonging to three types of chemical classes: terpenes, aromatics, and aliphatics (short-chain fatty acid) were used (supplementary table S1, Supplementary Material online). For functional studies of OR42 orthologs from different species, six odorants including PAA, benzaldehyde, (*E*)-2-hexen-1-al, cinnamaldehyde, 2-phenylethanol, and heptanal were used.

### Phylogenetic Analysis

The phylogenetic analysis was performed using 461 ORs from eight lepidopteran species including *H. armigera* (Liu et al. 2012; Zhang et al. 2015; Guo et al. 2018), *S. littoralis* (Jacquin-Joly et al. 2012; Poivet et al. 2013), *H. assulta* (Zhang et al. 2015), *Ostrinia furnacalis* (Yang et al. 2015), *C. suppressalis* (Cao et al. 2014), *B. mori* (Tanaka et al. 2009), *G. molesta* (Li et al. 2015), and *M. sexta* (Koenig et al. 2015). Alignments of amino acid sequences were performed using MAFFT (https://www.ebi.ac.uk/Tools/msa/mafft/). The tree was constructed using RAxML version 8 with the Jones–Taylor–Thornton amino acid substitution model (JTT) and 1,000 bootstrap replicates to assess node support. The main ligand of functional ORs of each species (*H. armigera*, Liu et al. 2013; Chang et al. 2016; *S. littoralis*, Montagné et al. 2012; de Fouchier et al. 2015, 2017; *H. assulta*, Chang et al. 2016; Cui et al. 2018; and *B. mori*, Nakagawa et al. 2005; Anderson et al. 2009; Tanaka et al. 2009) were mapped onto the phylogenetic tree.

The selective pressure acting on the OR sequences of each clade were calculated using the CODEML program implemented in the PAML 4.9 package that estimates ratios of the normalized nonsynonymous (d*N*) to synonymous (d*S*) substitution rate (*ω*) (Yang 2007). The OR genes with sequences less than 380 amino acid residue were removed when performing calculation. Clades L and O were excluded form analysis because only one gene remained after removing genes with short sequence. For the rest 21 clades, the codon sequences in each clade were aligned using ClustalW procedure and a maximum likelihood phylogenetic tree was reconstructed with MEGA X software separately (Kumar et al. 2018). The substitution rate (*ω*) of each lineage was calculated in CodeML procedure with Site model Model 0: one-ratio.

### Homozygote Mutant Construction by CRISPR/Cas9

The construction of HarmOR42-deletion mutants was performed according to previous reports (Chang et al. 2017; Wang et al. 2018). Cas9 protein originated from ThermoFisher (GeneArt Platinum Cas9 Nuclease, ThermoFisher Scientific, Pittsburgh, PA). Two target sites for single guide RNAs (sgRNA 1: 5′-GAACAATTCAAGATCTACACTGG-3′ and sgRNA 3: 5′-GCATCTGTTATCTTACACATGGG-3′) were chosen on the first exon of the HarmOR42 gene according to the manufacturer’s instructions (GeneArt Precision gRNA Synthesis Kit, ThermoFisher Scientific). sgRNAs were prepared by PCR assembly. First, the DNA template of each sgRNA was generated by using synthetic forward and reverse oligonucleotides with the Tracr Fragment + T7 Primer Mix. Forward strand oligonucleotides consisted of the universal forward primer (5′-TAATACGACTCACTATAG-3′) and the gene-specific target oligonucleotide. Similarly, the reverse strand oligonucleotides consist of the universal reverse primer (5′-TTCTAGCTCTAAAAC-3′) and with the gene-specific target reverse oligonucleotide. PCR assembly and in vitro transcription (IVT) were conducted according to the manufacturer’s instructions. After generating sgRNAs by IVT, the DNA template was removed by DNase I digestion, and sgRNAs were purified by using the gRNA Clean Up Kit (ThermoFisher Scientific, Pittsburgh, PA).

Freshly laid eggs (within 30 min of oviposition) were used for microinjection. About 1 nl of the Cas9/two target gRNA complex was injected into each egg using a FemtoJet and InjectMan NI 2 microinjection system (Eppendorf, Hamburg, Germany). The Cas9/gRNA complex consisted of 150 ng/μl of sgRNA1, 150 ng/μl of sgRNA3, and 150 ng/μl of Cas9 protein. Injected eggs were incubated at 26 ± 1 °C and 65 ± 5% RH for 3–4 days until hatching.

The female or male adults of the G0 generation from the microinjected eggs were hybridized with wild-type adults to generate F1 individuals. Once the F1 moths emerged from pupae, 96 individuals were randomly chosen for the detection of deletion mutants. Mid legs were used for genomic DNA extraction and PCR reactions were conducted to amplify a genomic fragment of HarmOR42, using gene-specific primers. Based on the HarmOR42 genomic sequence, the size of the PCR amplified fragment was expected to be 829 bp in wild-type individuals, and 600 bp in deletion mutants. F1 heterozygous mutants were first screened by agarose gel electrophoresis, based on the presence of two bands, and the genotype was further confirmed by DNA sequencing. F1 heterozygous mutants with the same genotype were crossed to generate F2, among which 25% were expected to be homozygote mutants with a large deletion present in the genome. F2 homozygous mutants were detected via the same procedure (gel detection and sequencing confirmation) and used for the following experiments.

### EAG Assays

The antennae of 2- to 3-day-old virgin male and female moths were cut at the base of the flagellum. After removing the tip, one antenna was inserted between two glass electrodes filled with 0.1 M KCl solution. In each test, a 10-μl solution of each odorant was added onto a piece of filter paper (0.5 cm × 0.5 cm) and then inserted into a Pasteur pipette. About 100 ng/μl stock solutions of individual odorants and different doses of the mixture IAC were prepared with paraffin oil. A continuous airflow of 30 ml/s was produced by a stimulus controller (CS-55, Syntech, Kirchzarten, Germany). Odor stimulation was controlled by a puff of purified air (0.2 s at 10 ml/s airflow) from the CS-55. EAG signals were amplified with a 10× AC/DC headstage preamplifier (Syntech) and further acquired with an Intelligent Data Acquisition Controller (IDAC-4-USB, Syntech) (Cao et al. 2016). The signals were recorded, monitored, and analyzed using Syntech EAG-software (Syntech, Germany). The EAG response values for each compound were calculated by subtracting the value of the same antennae corresponding to a solvent blank of paraffin oil.

### Behavioral Experiments

For adults, behavioral experiments were performed in a two-choice olfactometer according to a previous study with some modifications (Del Socorro et al. 2010). The olfactometer apparatus consisted of a main-chamber (60 cm × 30 cm × 25 cm) and two-choice subchambers under the main-chamber (20 cm × 15 cm × 25 cm) (fig. 6*A*). A metal gauze funnel (10 cm diameter) was inserted between the main-chamber and the subchambers. A hole at the bottom of the funnel allowed the moth to enter the subchamber. Clean humidified air flows (15 l/min) entered each subchamber and were exhausted by an exhaust fan. All the experiments were performed during the moth scotophase at 27 ± 1 °C. For each run, odorant and solvent (paraffin oil) were respectively added into cotton swabs that were mounted at the bottom of each subchamber. Thirty moths (either all males or all females) were introduced into the main-chamber with covered entrances (10 cm diameter each) of the subchambers with round metal gauze for acclimation. The gauze pieces were removed 10 min later. After 10 h, the number of moths in each subchamber was recorded. Four to six replicates were conducted for each odorant and each sex. A selection ratio (SR) was calculated using the formula: SR = *T*/(*T* + *C*), where *T* represents the number of moths that entered the test subchamber and *C* represents the number of moths that entered the control subchamber. Odorants used in all tests were prepared with paraffin oil. A quantity of 0.01 mg PAA was applied. For IAC, 0.1% IAC was prepared in paraffin oil, and 50 μl were applied for the test. The results were statistically analyzed using a χ^2^ test.

For assessing larval behavior to PAA, the experiments were performed in closed plastic Petri dishes of 15 cm diameter (fig. 6*D*) as in Poivet et al. (2013) with some modifications. To ascertain the choice zone, we tracked the behavior trajectory of larvae to a regular diet. A piece of regular diet nutrition was placed at one of the opposite ends of the dish along the diameter. Ten third instar larvae were placed in the middle of the dish. The location of larvae was marked every 10 min for 1 h. Four replications were performed. Most of the larvae made choices within 40 min, so this time point was selected for choice statistics. For the choice behavior test, PAA and paraffin oil (solvent) were respectively added onto a piece of 1 × 1 cm filter paper and the papers were arranged at opposite ends. The two-choice zones were delineated by 4-cm radius half circles centered at each of the filter papers. The choice indexes (CI) were calculated using the formula: CI = (*P* − *C*)/*T*, where *P* represents the number of larvae that entered the PAA choice zone, *C* represents the number of larvae that entered the solvent zone, and *T* represents the total number of larvae in the test (ten larvae). We first determined the efficient test dose using three different dilutions of PAA (0.1, 1, and 10 μg) against wild-type larvae. The CI of larvae to PAA was compared with a blank (solvent on each side of the dish). Wild-type larvae exhibited the most positive choice to PAA at the dose of 1 μg. Therefore, we used 1 μg of PAA to test the choice behavior of mutant larvae. Ten replicates were performed for each test.

### Data Analysis

Graphs and data statistics of OR response spectra, dose responses, EAG, and behavioral experiments were generated by Prism 7 (GraphPad Software, La Jolla, CA). A heat map was generated in Excel 2010 (Microsoft Corporation, WA). Tuning breadth graphs were generated according to the response of each OR to 67 odorants by putting the largest response in the middle and with other responses descending on both sides. For tuning breadth analysis, the kurtosis values (*K*) were calculated by SPSS 22 (SPSS Inc., Chicago, IL) and were used to define the tuning spectra width, where smaller *K* values represent broader tuning spectra (fig. 1*B*). PCA and hierarchical cluster analysis were conducted with PAST 3 software by using the mean response value of ORs to each odorant according to the description by Hallem and Carlson (2006). PCA was performed using the variance–covariance matrix, and hierarchical cluster analyses were conducted using Ward’s method and Euclidean similarity index.

## Supplementary Material

Supplementary data are available at *Molecular Biology and Evolution* online.

## Supplementary Material

msaa300_Supplementary_Data
