# Supplementary figures and images for: Odorant Receptors for Detecting Flowering Plant Cues Are Functionally Conserved across Moths and Butterflies
Source: Mol Biol Evol. 2020 Nov 24;38(4):1413–27. doi: 10.1093/molbev/msaa300 (PMC8042770; doi:10.1093/molbev/msaa300)

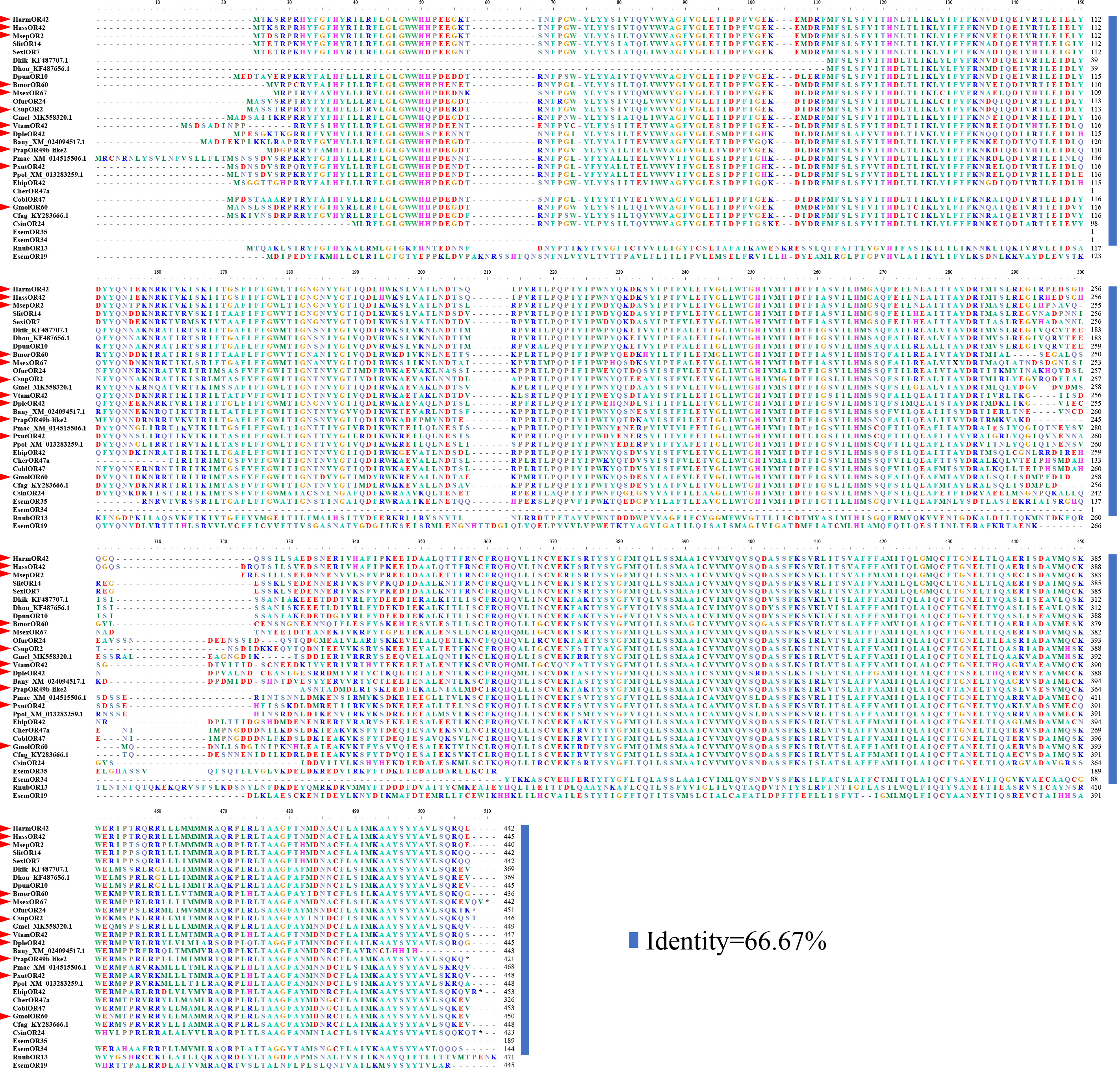

Supplement: msaa300_Supplementary_Data [file msaa300_supplementary_data.zip › supplementary fig. 2-final.jpg]
